# Irreversibility of Pulmonary Fibrosis

**DOI:** 10.14336/AD.2021.0730

**Published:** 2022-02-01

**Authors:** Qing Yang Yu, Xiao Xiao Tang

**Affiliations:** ^1^State Key Laboratory of Respiratory Disease, National Clinical Research Center for Respiratory Disease, National Center for Respiratory Medicine, Guangzhou Institute of Respiratory Health, The First Affiliated Hospital of Guangzhou Medical University, Guangzhou, China.; ^2^Guangzhou Laboratory, Bio-island, Guangzhou, China

**Keywords:** pulmonary fibrosis, irreversibility, pathogenesis, lung

## Abstract

Pulmonary fibrosis, a kind of terminal pathological changes in the lung, is caused by aberrant wound healing, deposition of extracellular matrix (ECM), and eventually replacement of lung parenchyma by ECM. Pulmonary fibrosis induced by acute lung injury and some diseases is reversible under treatment. While idiopathic pulmonary fibrosis is persistent and irreversible even after treatment. Currently, the pathogenesis of irreversible pulmonary fibrosis is not fully elucidated. The known factors associated with the development of irreversible fibrosis include apoptosis resistance of (myo)fibroblasts, dysfunction of pulmonary vessel, cell mitochondria and autophagy, aberrant epithelia hyperplasia and lipid metabolism disorder. In this review, other than a brief introduction of reversible pulmonary fibrosis, we focus on the underlying pathogenesis of irreversible pulmonary fibrosis from the above aspects as well as preclinical disease models, and also suggest directions for future studies.

Fibrosis is a pathological change manifested as aberrant tissue repair after chronic inflammatory injury, resulting in excessive synthesis of extracellular matrix (ECM) and replacement of normal parenchyma by ECM such as collagen. With continuous accumulation of ECM, organ fibrosis leads to structural damage, dysfunction and eventually organ failure [1]. Fibrosis occurs in many organs, including heart, kidney, liver and lung [2].

Pulmonary fibrosis, a type of terminal pathological change in the lung, is induced by chronic repetitive alveolar injury of various causes (including heredity, infection and environmental exposure, etc.), leading to excessive ECM deposition and accumulation. Some types of pulmonary fibrosis are reversible, while some others are not. Idiopathic pulmonary fibrosis (IPF) is the most common fibrotic lung disease with progressive and irreversible development [3, 4]. In contrast, pulmonary fibrosis induced by drug or acute lung injury seems reversible after systematic treatment [5-9]. The specific concepts of irreversible and reversible pulmonary fibrosis as well as the causes of their (ir)reversibility remain undefined. Thus, exploring the pathophysiological mechanisms of these two different types of pulmonary fibrosis is significant for the treatment. In this article, we review the potential concepts of irreversible pulmonary fibrosis and the underlying pathogenesis. Also, we propose future directions for the preclinical studies of pulmonary fibrosis.

## Currently proposed criteria of irreversible pulmonary fibrosis

A number of interstitial lung diseases (ILDs) and rheumatic diseases develop pulmonary fibrosis to a certain extent. These non-IPF fibrotic lung disease phenotypes may be partially stabilized and reversed after treatment [10]. But most pulmonary fibrosis is progressive and cannot be effectively alleviated even after aggressive treatment.

**Table 1:** Inclusion criteria for patients with PF-ILD in clinical practice.

Project	Inclusion criteria	Ref.
Nintedanib in PF-ILD	•Relative decline of FVC predicted ≥10% •Relative decline of FVC predicted between 5% to 10% with worsening respiratory symptoms •Relative decline of FVC predicted between 5% to 10% with extending fibrosis on HRCT •Worsening respiratory symptoms with extending fibrosis on HRCT •HRCT features of fibrotic lung diseases and fibrosis extended ≥10% •FVC ≥45% predicted •*D*LCO ≥30%—<80% of the predicted (HRCT signs must be assessed by experienced thoracic radiologist)	[12, 13]
Pirfenidone in unclassifiable PF-ILD (NCT03099187)	•Absolute decline of FVC >5% predicted or worsening symptoms not due to cardiac, pulmonary, vascular or other causes within the previous 6 months •Extending fibrosis >10% on HRCT •FVC ≥45% predicted, *D*LCO ≥30% predicted, FEV_1_/FVC ratio ≥0.7 •6MWD ≥150m (HRCT signs must be assessed by experienced thoracic radiologist)	[14, 15]
Pirfenidone for progressive, non-IPF lung fibrosis (RELIEF; EudraCT 2014-000861-32)	•Absolute annual decline of FVC ≥5% within 6 to 24 months	[16]
Antifibrotic drugs in non-IPF PF-ILD	•Relative decline of FVC ≥10% •Relative decline of *D*LCO ≥15% •Worsening symptoms •Worsening HRCT signs accompanied by a relative decrease of FVC ≥5-<10% (HRCT signs must be assessed by experienced thoracic radiologist)	[17]
Characteristics and outcomes of PF-ILD other than IPF (PROGRESS; NCT03858842)	HRCT shows >10% fibrosis areas and meets the following criteria for 2 years: •Relative decline of FCV ≥10% with or without clinical deterioration •Relative decline of FCV in 5%-10% with deteriorating respiratory symptoms •Relative decline of FVC in 5%-10% associated with extending fibrosis on HRCT •Extending fibrosis on HRCT with worsening respiratory symptoms (HRCT signs must be assessed by experienced thoracic radiologist)	[18]
Pirfenidone for Progressive Fibrotic Sarcoidosis (PirFS) (NCT03260556)	•Pulmonary function testing with CPI score ≥ 40 •HRCT shows the fibrosis signs >20% •Stable prednisone therapy for at least two months and no change in other immunosuppressives in the two months (HRCT signs must be assessed by experienced thoracic radiologist)	-
Nintedanib in Progressive Pneumoconiosis Study (NiPPS) (NCT04161014)	•HRCT shows diffuse fibrosing extent >10% in lung •FVC ≥45% predicted and *D*LCO >30% predicted (HRCT signs must be assessed by experienced thoracic radiologist)	-
Allogeneic Mesenchymal Stem Cells in Rapidly Progressive Interstitial Lung Disease (NCT02594839)	•Interstitial lung disease is diagnosed based on: ■Clinical symptoms >12 months duration ■Histologically diagnosed or HRCT features of interstitial pneumonia •FVC ≥40% predicted and *D*LCO ≥20% •Decline of 10% in FVC (L) and *D*LCO during the last 12 months (HRCT signs must be assessed by experienced thoracic radiologist)	-

*D*LCO: diffusing capacity of the lung for carbon monoxide; FEV_1_: forced expiratory volume in 1 second; FVC: forced vital capacity; HRCT: high-resolution CT; PF-ILD: progressive-fibrosing interstitial lung disease; 6WWD: 6 min walk distance.

These progressive pulmonary fibrosis phenotypes are generally known as “progressive fibrosing interstitial lung disease” (PF-ILD), that is irreversible or persistent pulmonary fibrosis [11]. Currently, there is no unified standard or definition of irreversible pulmonary fibrosis. Although some inclusion criteria for patients with PF-ILD have been formulated in clinical practice, the detailed index of these criteria is imperfect and not unified (Table 1) [12-18]. Cottin et al. and George et al. considered that patients with progressive pulmonary fibrosis must meet the conditions that the pulmonary function parameters, CT imaging manifestations and clinical symptoms of patients continue to deteriorate even after appropriate management. According to several clinical trials, they proposed the criteria of PF-ILD (Table 2) [11-13, 19, 20]. On the contrary, if the conditions of patients with pulmonary fibrosis are stabilized or even reversed after aggressive treatment, the pulmonary fibrosis phenotype can be considered as reversible.

**Table 2:** Suggested criteria or definitions of progressive fibrosis in clinical practice.

Suggested inclusion criteria 1 for clinical practice	Ref.
Patients meeting any of the following criteria within a 24-month period may have PF-ILD: •Relative decline of ≥10% in FVC •Relative decline of ≥15% in *D*LCO •Worsening symptoms or worsening HRCT signs accompanied by a relative decline of FVC ≥5-<10% (HRCT signs must be assessed by experienced thoracic radiologist)	[11]
Suggested inclusion criteria 2 for clinical practice	Ref.
Patients excluded the alternative explanations such as respiratory tract infection and meeting any of the following criteria can be considered to have PF-ILD: •Relative decline of 10% or more in FVC over 24 months despite treatment •Relative decline in FVC of 5% or more with decline in *D*LCO of 15% or more over 24 months despite treatment •Relative decline in FVC of 5% or more with increased fibrosis on HRCT over 24 months despite treatment •Relative decline in FVC of 5% or more with progressive symptoms over 24 months despite treatment •Progressive symptoms with increased fibrosis on HRCT over 24 months despite treatment (HRCT signs must be assessed by experienced thoracic radiologist)	[19]

CPI: Composite Physiologic Index; *D*LCO: diffusing capacity of the lung for carbon monoxide; FVC: forced vital capacity; HRCT: high-resolution CT; PF-ILD: progressive-fibrosing interstitial lung disease.

## Reversibility in pulmonary fibrosis

### Cases of reversible pulmonary fibrosis

Other than few clinical cases, pulmonary fibrosis induced by acute injury, viral infection, and some chronic non-IPF ILD (including chronic hypersensitive pneumonia, connective tissue disease-related interstitial lung disease and non-specific interstitial pneumonia) could be alleviated or even be cured, and pulmonary function of these patients was improved after treatment [8, 9, 19]. Besides, drugs like bleomycin (BLM), methotrexate and nitrofurantoin have certain pulmonary toxicity, leading to interstitial pulmonary fibrosis [21-23]. And lung fibrosis caused by drug toxicity is reversible. Drug induced-fibrotic lesions and lung function are substantially improved after drug withdrawal and the following medication [24, 25].

### Fundamental mechanisms of pulmonary fibrosis resolution

Most reversible fibrotic lung diseases are secondary to lung injury caused by infection or drug toxicity. Once the hazard is eliminated, lung fibrosis gradually resolves with treatment. This is also the case in the animal disease models. Almost all the animal models of pulmonary fibrosis have the characteristics of spontaneous resolution.

The BLM-induced pulmonary fibrosis animal model is widely used in the studies owing to the simple modeling method, low cost and obvious fibrosis lesion, etc. [26]. BLM causes apoptosis of alveolar epithelia by inducing DNA break and oxidative stress. The cytotoxic effects then result in acute lung injury and excessive inflammation. Finally, fibrosis occurs as a result of inflammation-induced fibroblast activation and ECM deposition [27, 28]. In general, 28 days after BLM injury, the fibrosis lesion gradually subsides and is close to normal at the end [27]. However, the mechanisms underlying spontaneous resolution in the BLM-induced animal model of pulmonary fibrosis are unspecified.

Elimination of matrix-producing cells, clearance of collagen matrix and regeneration of normal tissue are three important criteria necessary for fibrosis resolution [29]. Without these three elements, spontaneous resolution of pulmonary fibrosis in animal models cannot happen.

Activation of lung fibroblasts can be spontaneously reversed in some animal models [29]. Skeletal muscle terminal differentiation regulator MyoD modulates fibroblast (de)differentiation [30]. TGF-β up-regulates MyoD through ALK5 signaling, promoting fibroblast differentiation. While mitogen related factors also promote myofibroblast dedifferentiation by down-regulating MyoD through activating ERK1/2 MAPK and CDKs signaling [31]. The expression of MyoD is age-related. Aging is beneficial to the increase of MyoD, which maintains the phenotype of myofibroblasts. In the young BLM-induced mice, MyoD is significantly increased in fibrosis stage, while down-regulated in resolution stage, promoting the dedifferentiation of myofibroblasts. However, MyoD expression in the aged BLM-induced mice is consistently increased throughout the whole disease course, not showing fluctuant expression as in the young BLM-induced mice [32].

Apolipoprotein E (ApoE) is highly expressed in fibrosis stage of BLM-induced pulmonary fibrosis mice. Nevertheless, ApoE has no effect on the development of fibrosis, but on the regression of fibrosis. It binds to collagen I and mediates collagen phagocytosis via an apolipoprotein E receptor, low-density lipoprotein receptor associated protein 1 (LRP1), promoting resolution of pulmonary fibrosis [33].

In addition, genes related to lung development (such as FGF10) are markedly increased in the resolution stage of BLM-induced pulmonary fibrosis [34, 35]. The temporary up-regulation of FGF10 in resolution stage may play a key role in resolving fibrosis lesions and alveoli regeneration. In contrast, a stable and low expression of FGF10 is found in lung tissue from IPF patients [36].

Gene spectrum data of BLM-induced mouse model at different sampling time points showed that fibrosis-related genes and signaling, such as ECM remodeling, inflammatory response and Wnt signaling pathway, are up-regulated in the inflammatory and fibrosis stages. In the resolution stage, these genes are decreased, while those related to cell cycle and transcriptional regulation are up-regulated [37]. Up-regulation of these genes in resolution stage may inhibit the pro-fibrotic genes, thus inducing fibrosis resolution [38].

Therefore, as long as collagen phagocytosis and lung regeneration regulation are not fundamentally damaged, even though the massive alveoli are impaired and ECM is deposited, the fibrosis lesion will finally resolve.

## The mechanism underlying progressive development of irreversible pulmonary fibrosis

IPF, the most common progressive pulmonary fibrosis disease, is recognized as absolutely irreversible [39]. Besides IPF, some above-mentioned non-IPF chronic ILD may lead to irreversible fibrosis due to various unknown reasons [40]. These different irreversible fibrotic lung diseases seem to have similar symptoms and pathogenesis [41, 42]. Owing to the unclear etiology, occult pathogenesis, and lack of relevant animal models, it is difficult to elucidate why and how irreversible pulmonary fibrosis happens.

Recently, researchers found that repetitive intratracheal instillation of BLM in young mice or a single dose of BLM in aged mice shows persistent pulmonary fibrosis without spontaneous resolution [43-45]. These models provide grounds for the pathogenesis studies of persistent pulmonary fibrosis.

**Figure 1.**
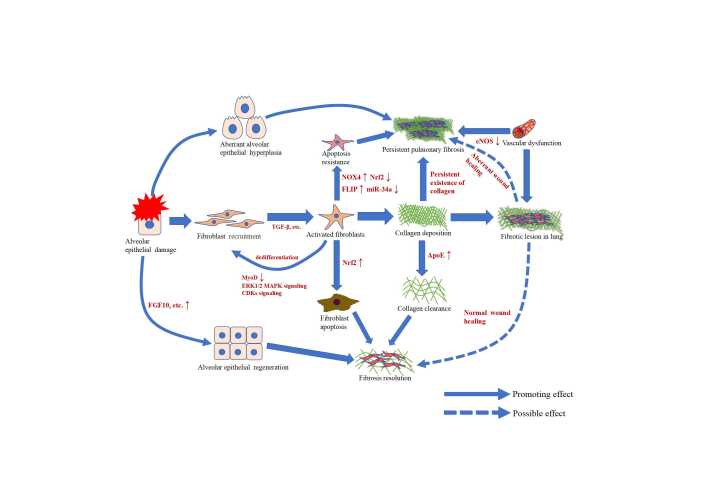
Schematic representation of the cellular events and basic mechanisms in persistent pulmonary fibrosis and fibrosis resolution. Alveolar epithelial damage causes recruitment of fibroblasts, which is activated by TGF-β, leading to collagen deposition and organ fibrosis. Fibrosis can be persistent, or eventually resolve via wound healing and lung regeneration. With normal collagen clearance and fibroblast apoptosis, fibrosis lesion in the lung is possible to resolve spontaneously. On the contrary, abnormal epithelial hyperplasia, fibroblast apoptosis resistance and collagen clearance failure caused by aberrant wound healing and pulmonary vascular dysfunction may lead to persistent pulmonary fibrosis.

In the following paragraphs, we take IPF as the main example of irreversible pulmonary fibrosis to discuss the pathogenesis (Table 3 & Fig. 1).

**Table 3:** Mechanisms of persistent pulmonary fibrosis.

	Underlying mechanisms	Model	Ref.
Apoptosis resistance of lung fibroblasts	Nox4-Nrf2 dysregulation in lung tissue impairs the redox capacity, endowing the myofibroblasts with the senescence and anti-apoptotic phenotype, which causes persistent pulmonary fibrosis.	BLM model of aged mice	[43]
	Fas signaling dysfunction caused by down-regulation of Fas or overexpression of anti-apoptotic protein induces lung fibroblasts resistant to apoptosis and retain the pro-fibrotic phenotype.	BLM model of Fas deficiency genetic mice	[55]
	FLIP induces IPF myofibroblasts to resist apoptosis and evade immune surveillance by activating NF-κB signaling.	IPF primary lung fibroblasts	[56]
	Dysregulated expression of miR-34a and FLIP reduces the susceptibility of myofibroblasts to lymphocyte-mediated apoptosis and leads to persistent pulmonary fibrosis.	MiR-34a dominant negative mice, C57BL/6J wild type mice	[57]
	HMGB1 released after lung injury induces apoptosis resistance of fibroblasts via activation of TLR4, leading to persistent pulmonary fibrosis.	Pulmonary fibrosis mouse model induced by radiation	[58]
Mitochondrial dysfunction	Mitochondrial dysfunction caused by stable suppression of PGC1α in IPF lung fibroblasts leads to activation of pro-fibrotic phenotype and promotes senescence of adjacent cells through paracrine manner, inducing persistent pulmonary fibrosis.	Aged *col1α1*-GFP transgenic mice	[62]
	Reduced expression of PINK1 induces mitochondrial dysfunction and release of profibrotic factors in ATIIs, increasing the susceptibility to lung fibrosis.	Wild type mice and genetic mice inoculated with MHV68; genetic mice treated with BLM	[66]
Pulmonary vascular dysfunction	Deficiency of eNOS caused by loss of endothelial phenotype and pulmonary vascular dysfunction leads to sustained fibroblast activation, resulting in persistent pulmonary fibrosis.	BLM model of aged mice and young eNOS^-/-^ mice	[72]
	Chronic repeated injury suppresses the CXCR7 expression and promotes macrophage recruitment. The recruited macrophages stimulate PECEs to increase Notch ligand Jagged 1, which then elicits sustained activation of Notch signaling in perivascular fibroblasts, promoting persistent pulmonary fibrosis.	Mouse model of repeated BLM instillation	[73]
Aberrant epithelial hyperplasia	The deficiency of Nedd4-2 enhances MUC5B expression by increasing surface expression and activity of ENaC in airway epithelia cells, inducing progressive pulmonary fibrosis via impaired mucociliary clearance and dysregulation of TGF-β signaling.	Conditional deletion of Nedd4-2 genetic mice	[91]
	Airway mucociliary dysfunction caused by high concentration of MUC5B in airways may be highly correlated with the persistent development of pulmonary fibrosis.	Mouse model of repeated BLM instillation	[94]
Lipid metabolic disturbance	ApoE binds to collagen I and mediates collagen phagocytosis via low-density lipoprotein receptor associated protein 1 (LRP1), promoting resolution of pulmonary fibrosis. While loss of ApoE leads to dysfunction of collagen phagocytosis, inducing persistent fibrosis.	ApoE^-/-^ mouse model	[33]
Autophagy dysfunction	Autophagy dysfunction in IPF lung fibroblasts induces persistent activation of mTOR, which contributes to the apoptosis resistance of lung fibroblasts.	Primary human lung fibroblasts	[107]
	Loss of autophagy gene ATG7 in endothelial cells induces EndMT and activates the TGF-β signaling pathway, aggravating pulmonary fibrosis.	EC-ATG^-/-^ mice	[108]

ApoE: Apolipoprotein E; BLM: bleomycin; CXCR7: chemokine (C-X-C motif) receptor 7; eNOS: endothelial nitric oxide synthase; FLIP: FLICE like inhibitory protein; HMGB1: high mobility group box 1 protein; LRP1: lipoprotein receptor associated protein 1; MUC5B: Mucin 5B; mTOR: the mammalian target of rapamycin; NOX4: NADPH oxidase 4; Nrf2: NFE2-related factor 2; PECEs; PGC1α: Peroxisome proliferator activated receptor gamma co-activator 1-alpha; PINK1: PTEN-induced putative kinase 1; TLR4: Toll like receptor 4.

### Apoptosis resistance of fibroblasts may be the key of irreversible development in pulmonary fibrosis

(Myo)fibroblasts play a significant role in wound healing. During wound healing, fibroblasts are recruited to the injured area by epithelial damage induced inflammation and differentiate into myofibroblasts induced by TGF-β. Normally, myofibroblasts gradually undergo apoptosis with wound healing. However, in the pathological state, persistent activation of (myo)fibroblasts leads to excessive scar hyperplasia and organ fibrosis [46, 47]. An altered level of apoptosis resistance in IPF (myo)fibroblasts results in their sustained activation and persistent pulmonary fibrosis [48].

Reactive oxygen species (ROS) related factor NADPH oxidase 4 (Nox4) has been evidenced to promote pulmonary fibrosis. Nox4 expression is markedly increased in the lung fibroblasts from IPF patients and BLM-induced pulmonary fibrosis mice [49, 50]. In the fibrotic lung, increased Nox4 induces lung fibroblasts to transform into senescent and apoptosis-resistant phenotype, aggravating pulmonary fibrosis. Generally, increased Nox4 can be neutralized in vivo by antioxidant factors, such as Nrf2, thereby alleviating oxidative stress [51]. But in IPF patients and the BLM-induced persistent fibrosis model of aged mice, Nrf2 expression in fibroblasts is significantly decreased and the antioxidant function is diminished [43, 52]. Nox4-Nrf2 dysregulation in lung tissue impairs the redox capacity, endowing the myofibroblasts with the senescence and anti-apoptotic phenotype, which causes persistent pulmonary fibrosis [43]. Targeting Nox4 and Nox4-related transcription factors restores fibrosis resolution in the aged mouse model [53].

However, Nox4 is not the only molecule that mediates fibroblast apoptosis in pulmonary fibrosis. Death factor Fas also regulates apoptosis of lung fibroblasts [54]. In IPF, dysfunction of Fas signaling caused by down-regulation of Fas or overexpression of anti-apoptotic proteins induces lung fibroblasts resistant to apoptosis. Lung fibroblasts with Fas deficiency retain the pro-fibrotic phenotype and persistently activate COL1A1 and α-SMA promoters [55]. FLICE like inhibitory protein (FLIP), an apoptosis regulator induced by cell death receptor, is highly expressed in myofibroblasts of IPF lungs. FLIP induces IPF myofibroblasts to resist apoptosis and evade immune surveillance by activating NF-κB signaling [56]. Besides, MiR-34a and FLIP negatively regulate each other. In pulmonary fibrosis, miR-34a expression is decreased while FLIP is upregulated, reducing the susceptibility of myofibroblast apoptosis, leading to development of persistent pulmonary fibrosis [57].

In the radiation-induced pulmonary fibrosis, pneumonocytes release high mobility group box 1 protein (HMGB1) after lung injury. HMGB1 may induce apoptosis resistance of fibroblasts via activating Toll like receptor 4 (TLR4) by NF-κB and PI3K/Akt signaling, leading to persistent pulmonary fibrosis [58].

### Mitochondrial dysfunction contributes to irreversible pulmonary fibrosis

Mitochondrial dysfunction is an important pathological feature in pulmonary fibrosis [59]. Peroxisome proliferator activated receptor gamma co-activator 1-alpha (PGC1α), a transcription co-activator, regulates mitochondrial biogenesis, oxidative phosphorylation and ROS detoxification [60]. It also mediates the resolution of fibrosis lesion [61]. Therefore, increased PGC1α expression in the late stage of BLM injury is beneficial to fibrosis resolution. In contrast, loss of PGC1α may lead to progressive development of pulmonary fibrosis. The stable suppression of PGC1α in IPF lung fibroblasts leads to decreased mitochondrial mass and function. Mitochondrial dysfunction activates pro-fibrotic fibroblast phenotype and promotes senescence of adjacent cells through paracrine manner, inducing persistent pulmonary fibrosis [62]. Similar as in pulmonary fibrosis, PGC1α expression in renal fibrosis and liver fibrosis is decreased in tubule epithelial cells and hepatocytes, respectively. Overexpressing PGC1α expression restores mitochondrial function and protects from fibrosis [63, 64].

Expression of PTEN-induced putative kinase 1 (PINK1), an aging-associated key regulator of mitochondrial function, is decreased in the aged lung and IPF lung [65]. Reduced PINK1 expression causes mitochondrial dysfunction in type II alveolar cells (ATIIs), leading to endoplasmic reticulum stress and mitophagy dysfunction. Besides, deficient expression of PINK1 in ATII induces release of profibrotic factors. These pathologic processes influence the susceptibility to lung fibrosis and may contribute to irreversible lung fibrosis [66].

### Dysfunctional pulmonary vessel offers fundamental environment for progressive development of pulmonary fibrosis

In the mature lung, pulmonary vessel carries blood for gas exchange and nutrient transport. Besides, pulmonary capillary endothelial cells (PCECs) release varieties of cytokines to support the development, regeneration and wound healing in lung [67]. Structure loss, vascular barrier dysfunction, and increased vascular permeability caused by chronic and persistent lung injury continuously recruit and activate fibroblasts, and eventually result in collagen deposition and pulmonary fibrosis [68]. Previous data showed that aberrant vascular remodeling and increased alveolar capillary permeability in fibrotic lungs may result from the imbalanced abundance of pulmonary vascular endothelial cells and progenitors, as well as the imbalance between profibrotic and antifibrotic cytokines. The degree of the increase in vascular permeability is associated with the prognosis of patients with IPF [68-71].

Generally, endothelial cells increase the expression of nitric oxide synthase 3 (NOS3), which synthesizes endothelial nitric oxide synthase (eNOS) following lung injury. ENOS promotes nitric oxide (NO) to bind and activate soluble guanylate cyclase (sGC), inactivating lung fibroblasts and facilitating fibrosis resolution [72]. The aged mouse model of persistent pulmonary fibrosis shows the same pulmonary vascular retrogression as the IPF patients. Degeneration of pulmonary vessel leads to decrease of vascular density, loss of endothelial phenotype and unable to encode *Nos3* by endothelial cells, promoting persistent pulmonary fibrosis [72].

PCECs are activated and then increased the expression of chemokine receptor CXCR7 after lung injury. CXCR7 inhibits epithelial-mesenchymal transition (EMT) and pulmonary fibrosis by blocking Jag1-Notch signaling, thereby protecting the alveolar epithelia from injury. Chronic injury induced by repetitive BLM instillation suppresses CXCR7 expression and promotes macrophage recruitment around vessel. The recruited macrophages stimulate PCECs to increase Wnt/β-catenin dependent Notch ligand Jagged 1, which then promotes persistent pulmonary fibrosis by sustained activation of Notch signaling in perivascular fibroblasts [73-75].

### Aberrant epithelial hyperplasia and dysfunction may contribute to persistent development of fibrosis

Histologically, the pathological changes of IPF are not just ECM replacement. Atypical epithelial cells usually exist in the fibrosis areas that losing the normal alveolar structure as well as some normal areas. These cells express the markers of ATIIs, proximal airway and submucosal glands in bronchi, resulting in ectopic hyperplasia of alveolar epithelia cells, so-called “epithelial bronchiolization” [76, 77]. It is still unclear where these ectopic epithelial cells come from and what their effects are in persistent pulmonary fibrosis.

A recent study showed that in the pneumonectomy-induced pulmonary fibrosis mouse model, lack of *cdc42* causes unsuccessful differentiation of ATII to type I alveolar epithelial cells (ATIs) and promotes formation of transitional cells (TCs) between ATII and ATI, inducing persistent fibrosis [78]. Alveolar TCs are necessary for the differentiation of ATII into ATI during lung regeneration and can be divided into early and late stages [79, 80]. During the early stage, TGF-β is highly activated and increases keratin (KRT)8/KRT18 in TCs; while deactivation of TGF-β promotes early-stage TCs to transdifferentiate into late-stage cells via down-regulating KRT8/KRT18. The late-stage TCs are beneficial to the differentiation of ATII into ATI [80, 81]. A large number of early TCs with high KRT8/KRT18 expression exist in the lungs of IPF patients and BLM-induced pulmonary fibrosis mice [80]. The early TCs gradually transdifferentiate into late TCs in the BLM mouse model, while persistently exist in the IPF lungs [80, 82].

Although the markers of human TCs and mouse TCs differ, these two types of TCs naturally show DNA damage and cell senescence, especially under stimulation of aberrant mechanical stretch [79]. The abnormal biomechanical system in fibrotic lung induces DNA damage and senescence of TCs, promoting vicious development of fibrosis [83].

TCs appear in both persistent and reversible pulmonary fibrosis. They transform to epithelia with resolution of fibrosis, while IPF TCs are stuck in a certain stage and fail to detach, thereby leading to pathological damage and a vicious circle of the organ [84]. In addition, recent single cell RNA sequencing data showed that TCs found in IPF lungs express epithelia cell markers as well as *COL1A1* and other pathologic ECM component, suggesting that TCs may promote collagen production and fibrosis progression in IPF [85].

Recently, it has been evidenced that the mutation of *MUC5B* rs35705950 non-risk alleles is the strongest genetic risk factor of IPF. MUC5B, highly expressed in the IPF lungs, is regulated by ERN2-XBP1S signaling [86-88]. Besides, complement C3, FOXA2 and Nedd4-2 also affect MUC5B expression in the fibrotic lung [89-91]. The E3 ubiquitin-protein ligase Nedd4-2 is involved in epithelial homeostasis. In lung fibrosis, Nedd4-2 deficiency enhances MUC5B expression by increasing surface expression and activity of ENaC on airway epithelia cells [91]. The previous data showed that the overexpressed MUC5B is located in the epithelia cells from honeycomb cysts zone in the IPF lungs, and the ectopic expression of MUC5B may enhance the honeycomb-like cyst formation [92, 93].

In the BLM-induced pulmonary fibrosis mouse model, high concentration of MUC5B in airways causes mucociliary dysfunction and enhances the severity of pulmonary fibrosis [94]. The ectopic expression of MUC5B may lead to more severe and irreversible fibrosis by increasing the sensitivity of ATIIs to BLM and promoting formation of honeycomb structures [93]. Over-secreted MUC5B may not only break the mucosal host defense but also damage ATIIs and interfere with alveolar repair. The damaged alveoli fail to be epithelialized and enhance the collapse and fibrosis of bronchial-alveoli units, eventually leading to idiopathic pulmonary fibrosis [95]. But the direct cytotoxic effect of MUC5B on ATIIs in pulmonary fibrosis remains indistinct. Further investigations need be carried out in the future.

### Lipid metabolic disturbance and irreversible pulmonary fibrosis

At present, a growing number of studies have preliminarily demonstrated that lipid metabolism disorder is related with the pathogenesis of pulmonary fibrosis [96]. Previous studies showed decreased levels of lipid metabolism related molecules such as elongation of long-chain fatty acids family member 6 (Elovl6) and stearoyl CoA desaturase 1 (SCD1) in IPF lungs. And fibrosis susceptibility is increased when these genes are suppressed in mice [97, 98]. Besides, a single dose of BLM instillation induces more severe pulmonary fibrosis in the ApoE^-/-^ mice and the fibrosis develops progressively and irreversibly [33].

Sequencing data showed that the genes and signaling pathways related to lipid metabolism are down-regulated in the lungs of IPF patients and the aged mice with BLM injury [99, 100]. The balance of lipid metabolism is important in maintaining structure and function of the alveolar epithelium. Excessive accumulation of cholesterol leads to alveoli collapse and alveolar injury [101]. Based on the above, lipid metabolism disorder may play a key role in persistent pulmonary fibrosis.

### Autophagy may contribute to irreversible pulmonary fibrosis

Autophagy, a cytoprotective mechanism, is important to maintain cellular homeostasis and modulate redox equilibrium. Altered autophagy has been observed in pulmonary fibrosis [102]. Studies have shown that decreased autophagy in fibroblasts and alveolar epithelia promotes pulmonary fibrosis [103].

Autophagy dysfunction, mainly induced by aging, is associated with cell apoptosis resistance [104]. Recent studies showed that insufficient autophagy promotes IPF development. Autophagy inhibition in IPF lung fibroblasts and alveolar epithelia induces activation of lung fibroblasts [105, 106]. Autophagy dysfunction in IPF lung fibroblasts induces persistent activation of the mammalian target of rapamycin (mTOR), leading to apoptosis resistance of lung fibroblasts and persistent pulmonary fibrosis [107].

Besides, impaired autophagic flux of lung endothelial cells (ECs) induces change of endothelial structure and affects progression of pulmonary fibrosis [108]. This study also showed that loss of autophagy gene ATG7 in ECs induces endothelial-to-mesenchymal transition (EndMT) and activates the TGF-β signaling pathway in vitro. Comparing with the wild type mice, more serious fibrotic lesions occur in the EC-ATG7^-/-^ mice, indicating that progressive lung fibrosis may be accompanied by the loss of ATG7 [108].

### Small airway may be the breeding site for irreversible pulmonary fibrosis

Although IPF has been studied for decades, its onset site is still a mystery. As early as 40 years ago, it was reported that small airway lesion occurs in IPF, which is considered as a disease of small airway and alveoli. This concept was verified by pulmonary function test and histological assessment [109, 110]. Small airway is regarded as a “quiet zone” in lung. The injury and disease may easily accumulate in small airway for years without being noticed [111]. In IPF, pathological change in small airway is an early feature [110].

Club cells are the major epithelial cells in the small airways [112]. In IPF, club cells that express the specific protein SCGB1A1 are absent in the abnormal small airways. Bronchiolization, the characteristic change in IPF lung, precedes the fibrotic process. The density of SCGB1A1^+^ club cells is negatively correlated with the degree of bronchiectasis and bronchiolization, whereas positively correlated with forced vital capacity (FVC) [113]. Therefore, absence of SCGB1A1^+^ cells in the pathologic small airway may indicate development of severe fibrosis. Also, an earlier study showed that club cells migrate to the alveolar regions and directly induce alveolar apoptosis in IPF lungs [114]. Therefore, we speculate that the small airway may be the origin site of IPF.

If microdamage initially appears in small airway, the pathological lesion is hard to be noticed. With the accumulation of lesions, disease will rapidly worsen and eventually lead to organ failure. This process is in line with the development of IPF [115]. Thus, pathological change in small airway may be one of the factors for the occult and persistent development of IPF.

## Conclusions and Perspective

**Figure 2.**
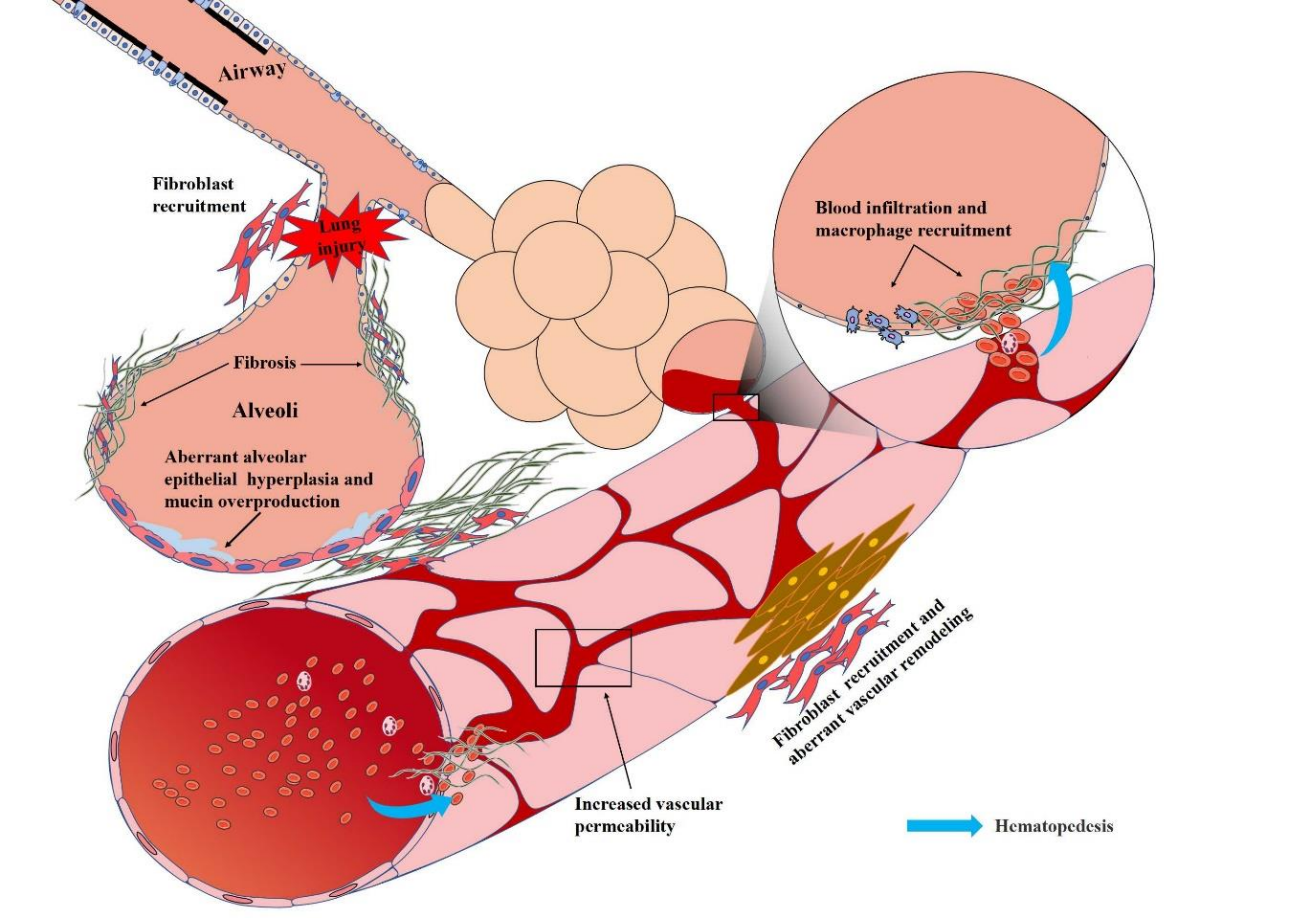
Possible pathogenesis of persistent pulmonary fibrosis. On the one hand, lung injury induces fibroblast recruitment, leading to collagen deposition and fibrosis. On the other hand, the incomplete differentiation of alveolar epithelia may result in aberrant alveolar epithelial hyperplasia and mucin overproduction, which may destroy the wound healing and aggravate pulmonary fibrosis. Besides, pulmonary vascular dysfunction caused by the loss of endothelia phenotype and high vascular permeability may induce aberrant vascular remodeling, which further enlarges the fibrosis lesion and results in the persistent and progressive development of pulmonary fibrosis.

Here we emphatically discuss the irreversible development of pulmonary fibrosis from aspects of clinical cases, animal models and pathogenesis. The apoptosis resistance of lung fibroblasts as well as dysfunction of mitochondrion, pulmonary vessel and lipid metabolism contribute to development of persistent pulmonary fibrosis. Besides, aberrant epithelial hyperplasia and cell autophagy potentially cause the irreversibility of pulmonary fibrosis. Also, we propose that small airway may be the origin site of persistent pulmonary fibrosis (Fig. 2).

Several pulmonary fibrosis phenotypes can be cured after treatment, while IPF and some non-IPF PF-ILD are progressive and incurable. Pulmonary fibrosis induced by acute lung injury is curable, probably because of the incomplete injury of epithelial regeneration and fibroblast apoptosis. As long as the normal stem cells exist, as well as wound healing and collagen degradation still work, fibrosis will gradually resolve. However, fibrosis resolution does not happen in PF-ILD, and the pathogenesis of persistent fibrosis remains unclear. As mentioned above, development of persistent pulmonary fibrosis involves a complex network. Therefore, targeting a certain class of cytokines or cell signaling may not be sufficient for disease remission [116].

Small airway damage in pulmonary fibrosis has been noticed for decades, whereas the relationship between the causes of injury in small airway and the initiation as well as sustained progression of pulmonary fibrosis is rarely studied. The main reason may be lack of systematic and continuous monitor methods for small airway damage in patients with PF-ILD. Besides, lack of suitable animal models and tissue models also makes it difficult to carry out studies on the small airway damage in pulmonary fibrosis. Development of relevant models and systematic studies based on small airway may be a key direction to explore the occult onset and persistent development of PF-ILD. Studies on small airway in pulmonary fibrosis may provide not only therapeutic targets, but also strategies for early diagnosis and monitoring of patients with PF-ILD.

Recently, organoids and cell models that modeling the pathological changes of IPF have been developed, including human induced pluripotent stem cell (iPSC)-derived air-liquid interface (ALI) model stimulated by IPF-relevant cocktail (IPF-RC) and the immortalized human small airway basal stem/progenitor cell line [117, 118]. These are expected to be preferred models for studies on small airway damage in pulmonary fibrosis.

Animal models of irreversible and persistent pulmonary fibrosis have been developed, including aged mice with single BLM instillation, young mice with repeated BLM instillation and some genetic mouse models. Nevertheless, these models also have limitations. Genetic mouse models cannot be widely used because of the gene specificity [27, 119]. The single dose of BLM-induced lung fibrosis in the aged mice is irreversible, however, it lacks IPF characteristic lesions such as airway epithelial hyperplasia [44]. Thus, although it takes a long time to establish, the mouse model of repeated BLM instillation may be the most advantageous model of persistent pulmonary fibrosis at present as it not only develops persistent fibrosis, but also represents UIP features, such as airway epithelial hyperplasia seen in IPF. This model is more consistent with the course and lesion of IPF [44, 45, 120].

These animal models of persistent pulmonary fibrosis are generally used for study of pathogenesis. However, few researchers systematically compare different kinds of models which are helpful to find target genes and biomarkers related to persistent pulmonary fibrosis. Furthermore, comparing the clinical symptoms, differentiation of histological features between reversible and irreversible pulmonary fibrosis, as well as exploring the mechanisms underlying persistent pulmonary fibrosis are of great significant for the early diagnosis and treatment of some occult pulmonary fibrosis, such as IPF.
